# Dietary diversity and associated factors among adult cancer patients attending treatment at Black Lion Specialized Hospital, Addis Ababa, Ethiopia

**DOI:** 10.1017/jns.2022.114

**Published:** 2023-01-12

**Authors:** Helen Girma, Amanuel Nana

**Affiliations:** 1 Menelik II Comprehensive Specialized Hospital, Addis Ababa City Administration, Addis Ababa, Ethiopia; 2 Department of Human Nutrition, Menelik II Medical and Health Science College, Kotebe University of Education, Addis Ababa, Ethiopia

**Keywords:** Dietary diversity, Ethiopia, People living with cancer, AOR, adjusted odd ratio, BLSH, Black Lion Specialized Hospital, COR, crude odd ratio, DD, dietary diversity, DDS, Dietary Diversity Score, IDDS, Individual Dietary Diversity Score, LMICs, low- and middle-income countries, SPSS, Statistical Package for Social Sciences

## Abstract

*Background:* Cancer has become a significant public health issue around the world and an increasingly important contributor to disease burdens. In countries like Ethiopia with high nutrient demands, people with chronic diseases like cancer are at a high risk of macro and micronutrient deficiencies. Therefore, the present study attempted to assess dietary diversity and associated factors among adult cancer patients attending treatment at Black Lion Specialized Hospital, Addis Ababa, Ethiopia. *Method and Materials*: Hospital-based cross-sectional study was conducted from 22 April 2021 to 22 May 2021 on 416 adult cancer patients at Black Lion Specialized Hospital (BLSH). A systematic random sampling technique was applied to select study subjects. Quantitative data were collected using a structured, pretested and interviewer-administered questionnaire. The questionnaire comprised the standard dietary diversity measurement tool, which was adopted from the Food and Technical Assistance (FANTA) then data were entered into EPI INFO software and analysed using Statistical Package for the Social Sciences (SPSS) version 25. Frequency, mean and standard deviation were used to describe variables. A binary logistic regression model was fitted to elicit factors associated with the dietary diversity of cancer patients and a *P*-value of less than 0⋅2 was used as a cut-off for further analysis. Logistic regression analysis with a 95 % confidence interval (CI) was estimated to measure the strength of association at *P* < 0⋅05. *Results:* The present study revealed that 61⋅5 % of patients had low dietary diversity. Being from a family size of five and more (AOR = 1⋅48, 95 % CI 1⋅28, 1⋅83), having no permanent income (AOR = 1⋅31, 95 % CI 1⋅15, 1⋅67), alcohol consumption (AOR = 3⋅97, 95 % CI 1⋅20, 13⋅1), not doing regular physical exercise (AOR = 1⋅83, 95 % CI 1⋅07, 3⋅12), lack of nutritional information (AOR = 2⋅23, 95 % CI 1⋅30, 3⋅82), poor nutritional knowledge (AOR = 1⋅84, 95 % CI 1⋅05, 3⋅25) and minimum meal frequency (AOR = 10⋅7, 95 % CI 5⋅04, 22⋅7) were factors associated with inadequate dietary diversity. *Conclusion*: The present study showed that the majority of cancer patients had low dietary diversity, suggesting that they are highly vulnerable to micronutrient deficiencies. Therefore, efforts should be strengthened to improve patients’ income level, access to nutrition information and nutritional knowledge.

## Background

Cancer has become a significant public health issue around the world and an increasingly important contributor to disease burdens. Globally, 19⋅3 million cases and 10 million cancer deaths were registered in 2020^(1)^. Low- and middle-income countries (LMICs) account for roughly 57 % of all new cancer cases worldwide^(1)^. Although cancer is a low priority in Africa's research and healthcare systems, its incidence and fatality rates are rising^(2)^. The current cancer prevalence in Ethiopia is 77 352 of the 114 963 583 total population^(4)^. About 60 960 new cases are reported each year, accounting for about 5⋅8 % of all national mortality^(5)^. Based on Ethiopia's national cancer control plan for 2016–2020, this trend is expected to continue in the coming years, due to changes in diet and lifestyle^(4)^.

Cancer and dietary issues are common, interrelated and exacerbate each other in a vicious cycle. Cancer alters one's nutritional status by increasing demands, decreasing food intake and interfering with nutrient absorption and metabolism^(6)^. Poor nutrition, on the other side, has an impact on disease progression, morbidity and mortality^(5)^. Micronutrients are also essential for an optimal immune function to be maintained. However, the availability of many vitamins and trace minerals is deteriorating in cancer patients, both at the time of diagnosis and after treatment begins^(6)^.

Micronutrient deficiency due to poor quality, non-diversified diet or low-quality monotonous diet is a serious problem among cancer patients^(7)^. Cancer patients with poor diet quality have a 65 % high risk of dying of cancer as compared to cancer patients having a balanced diet. In addition, utilisation of a diversified diet (a diet containing all nutrient-rich food groups) among cancer patients remains low in resource-limited African countries^(8)^. Besides, individuals with inadequate micronutrient intake have a 26 % high risk of mortality^(9)^.

The aetiology of poor dietary diversity (DD) and inadequate nutrient intake in cancer patients is complex and multi-factorial. Consequently, a person's dietary diversity is influenced by a variety of factors, including age, place of residence, educational level and monthly income. Other important factors include the kind of cancer, the year of diagnosis, the type of treatment and the existence of treatment side effects^(10–12)^. Moreover, behavioural factors and dietary knowledge of patients about the importance of nutrition for the prognosis and quality of life play a role in poor dietary intake^(13)^.

Food-based strategies such as dietary diversification have been advocated as the main priority to meet micronutrient needs^(2)^. Dietary diversity score (DDS) is a qualitative measure of food intake that represents the nutritional adequacy of individual diets^(14)^. Even though measuring diet quality is multidimensional, DDS is the simplest and most feasible indicator of diet quality^(15)^. It is most commonly calculated by counting the number of food groups rather than the food products eaten. While the reference date can vary, it is usually the previous day or week. DD's ratings were positively associated with increased mean dietary micronutrient density and micronutrient dietary adequacy in adults^(16–18)^.

Cancer patients’ nutritional health can be impacted by a non-diversified diet and insufficient nutrient intake, which can reduce immunity and speed the progression of the disease, leading to higher morbidity and fatality rates. This may hinder poor countries like Ethiopia from entering the track to reach the national cancer control plan as well as the global target established by the United Nations Sustainable Development Goals (SDGs) of reducing the risk of premature death from non-communicable diseases including cancer by one-third by 2030^(4,19)^.

Morbidity and mortality related to poor diet quality in cancer patients are high^(8,9)^. To improve it, dietary diversification needs to be sustainably improved through addressing information and reduction of barriers to adherence. Even though Ethiopia's national nutritional policy suggests improving the nutritional service delivery for non-communicable diseases as one of its strategic objectives, its execution regarding cancer is still low and should further be supported with actual research findings^(20)^. Therefore, the present study attempted to assess dietary diversity and associated factors among adult cancer patients attending follow-up treatment at Black Lion Specialized Hospital (BLSH) since it is not well understood in the study area of interest and Ethiopia as well.

## Method and materials

### Study area and study period

A hospital-based cross-sectional study was conducted among adult cancer patients from 22 April 2021 to 22 May 2021 at BLSH (Black Lion Specialized Hospital). BLSH is the largest referral hospital in the country and is the main hub for cancer registry, early detection, prevention, routine therapy and palliative care in Addis Ababa.

### Participants

The source populations were all adult cancer patients attending treatment at BLSH. The study populations were adult cancer patients (≥18 years) who were attending treatment at BLSH during the study period. Cancer patients who were seriously ill, pregnant, or had taken in unusual special festivals during the last 24 h before data collection were excluded from the study.

### Sample size and sampling procedure

The minimum number of samples required for this study was estimated by using single population proportion formula considering a proportion of 50 %, a margin of error (0⋅05), critical value at 95 % confidence level (Z1−*ᾳ*/2 = 1⋅96) and non-response rate (10 %) giving the total sample size 422. The systematic random sampling technique was used to select study participants by taking the monthly average of 1769 to calculate the *K*th (*N*/*n* = 1769/422 = 4) interval. The first participant was selected by a lottery method and then every fourth participant were enrolled until the appropriate sample size was achieved.

### Data collection tools and procedures

Data were collected from the study subjects by using an interviewer-administered, structured and pretested questionnaire. The questionnaire comprised socio-demographic, behaviour, nutrition, disease and treatment-related questions, and a standard dietary diversity measurement tool adopted from the Food and Technical Assistance (FANTA)^(14)^. Nutritional knowledge assessment questions were developed from the general nutritional knowledge questionnaire and modified with cancer dietary guidelines^(21,22)^.

English version of the questionnaire was prepared, translated to the Amharic version and back to English by language translators and nutrition experts to check for consistency. The questionnaire was pretested on 5 % of the total sample patients at St. Paul's Oncology Center. Six data collectors and one supervisor were trained for one day on the aim of the study, about consent, and how to approach and collect the data. Daily monitoring and supervision were done and questionnaires were examined for completeness.

### Measurement of the dietary diversity score

Individual dietary diversity scores indicate the likelihood of a diet's micronutrient adequacy as a result of the use of nine food groups such as starch staples, dark green leafy vegetables, other vitamin-A-rich fruits and vegetables, other fruits and vegetables, legumes, nuts and seeds, meat and fish, organ meat, egg, and milk and milk products. As oils and fats do not make a substantial contribution to dietary micronutrient density these food classes are not used to estimate individual dietary diversity ratings^(23)^. However, oils and fat food groups are energy dense and essential for the absorption of fat-soluble vitamins. As a result, the proportion of study participants who consume this food group was also calculated on their own.

The dietary diversity score of participants was determined primarily by listing all food items consumed (both in and out of home) at breakfast then lunch and dinner while snacks were considered to be eaten before or after the main meal (Breakfast, Lunch and Dinner). Then participants who ate all the foods in each subgroup at least once in the previous 24 h scored one point and zero point if they never consumed food. The mean individual dietary diversity score (IDDS) was considered as a cut-off^(14,23)^. To minimise the day-of-the-week effect, authors considered the weekend and market day during dietary data collection.

The knowledge of the study participants was assessed by considering ten knowledge questions. The scores of knowledge were obtained by summation of responses to each question. Each question was given one mark if the answers were correct or favourable answers. Zero scores were given if the responses were wrong or unfavourable answers. Then, participants were classified to have poor knowledge if they correctly answer below mean and good knowledge if they correctly answer the mean^(24)^.

### Operational definition

- *Dietary diversity*: Number of individual food groups consumed over a 24-h period and can be classified as both low and high dietary diversity^(14)^.
- *Low dietary diversity*: When individuals consume four and less than four food groups (mean dietary diversity score)^(14)^.
- *High dietary diversity*: When individuals consume five and above food groups^(14)^.
- *Poor nutritional knowledge*: When individuals score less than seven questions out of ten knowledge assessment questions (below the mean)^(24)^.
- *Good nutritional knowledge*: When individuals score seven and more questions out of ten knowledge assessment questions (above the mean)^(24)^.

### Data management and analysis

All filled questionnaires were checked for completeness and entered into EPI INFO version 7.2 software. Then the data were transferred to SPSS Version 25 for analysis. Frequency, proportion, mean and standard deviation were used to summarise the findings. A binary logistic regression model was fitted to elicit factors associated with the dietary diversity of cancer patients and a *P*-value of less than 0⋅2 was used as a cut-off for further analysis. Logistic regression analysis with a 95 % confidence interval (CI) was estimated to measure the strength of association at *P* < 0⋅05. Hosmer and Lemeshow goodness-of-fit test was performed to check model adequacy, yielding a *P*-value of 0⋅35 and a model chi-square of 8⋅89.

## Result

### Socio-demographic and economic characteristics of the study participants

A total of 416 adult cancer patients participated in the study with a response rate of 98⋅5 %. Out of the total respondents, 260(62⋅5 %) were females and the mean (±sd) age of respondents was 46⋅2(±14⋅3) years. Two-hundred and seventy-eight (66⋅8 %) of the study participants were urban residents and 268(64⋅4 %) were married. The mean (±sd) family size of respondents was 4⋅88(±2⋅52) and above half 225(54⋅1 %) of cancer patients had a family size of five and more. One-hundred and sixteen (27⋅9 %) of study participants were unable to read and write and 132(31⋅7 %) had no permanent monthly income (Table 1).

**Table 1.** Socio-demographic and economic characteristics of adult cancer patients (≥18 years) at BLSH, Addis Ababa, Ethiopia, 2021 (*n* = 416)

Variables	Categories	Frequency	Percent
Sex	Male	156	37⋅5
	Female	260	62⋅5
Age	19–29	51	12⋅3
	29–39	93	22⋅4
	39–49	107	25⋅7
	≥50	165	39⋅6
Place of residence	Urban	278	66⋅8
	Rural	138	33⋅2
Educational status	Unable to read and write	116	27⋅9
	Read and write	46	11⋅1
	Elementary (Grades 1–8)	80	19⋅2
	Secondary (Grades 9–12)	92	22⋅1
	Collage and above	82	19⋅7
Employment status	Unemployed^a^	254	61⋅1
	Employed	162	38⋅9
Family size	<5	191	45⋅9
	≤5	225	54⋅1
Income	No permanent income	132	31⋅7
	≤3000 ETB	130	31⋅5
	>3000 ETB	154	36⋅8

a Housewives, student and those who stop working because of illness.

### Behavioural characteristics of the study participants

A larger proportion of adult cancer patients 398(95⋅7 %) were non-smokers and 346(83⋅2 %) participants had no alcohol-drinking habit. One-hundred and ninety-nine (47⋅8 %) had a habit of doing regular physical exercise (Table 2).

**Table 2.** Behavioural characteristics of adult cancer patients (≥18 years) at BLSH, Addis Ababa, Ethiopia, 2021 (*n* = 416)

Variables	Categories	Frequency	Percent
Cigarette smoking	Yes	18	4⋅3
	No	398	95⋅7
Drinking Alcohol	Not at all	346	83⋅2
	Once a week	35	8⋅4
	Twice a week	23	5⋅5
	Three or more times a week	12	2⋅9
Doing regular exercise^a^	Yes	199	47⋅8
	No	217	52⋅2

a A minimum of 150 min per week of moderate-intensity physical activity.

### Cancer treatment-related characteristics

More than one-third of patients 155(37⋅3 %) were diagnosed with cancer less than a year before this study and 177(42⋅8 %) were taking chemotherapy. Among the most common treatment-related side effects, 106(25⋅5 %) had problems of either chewing, eating or swallowing, 256(61⋅5 %) had a loss of appetite, 215(51⋅7 %) had nausea and 137(32⋅9 %) had vomiting (Table 3).

**Table 3.** Cancer treatment-related characteristics of adult cancer patients (≥18 years) at BLSH, Addis Ababa, Ethiopia, 2021 (*n* = 416)

Variables	Categories	Frequency	Percent
Year of diagnosis	<1 year	155	37⋅3
	1–3 years	148	35⋅6
	3–5 years	72	17⋅3
	>5 years	41	9⋅8
Type of treatment	Surgery	94	22⋅8
	Chemotherapy	177	42⋅8
	Radiotherapy	74	17⋅8
	Palliative care	51	12⋅3
	Combined therapy	18	4⋅3
Problems with chewing/ eating/ swallowing	Yes	106	25⋅5
	No	310	74⋅5
Loss of appetite	Yes	256	61⋅5
	No	160	38⋅5
Nausea	Yes	215	51⋅7
	No	201	48⋅3
Vomiting	Yes	137	32⋅9
	No	279	67⋅1

### Nutrition knowledge and information-related characteristics

Out of 416 participants, 269 (64⋅7 %) had good nutritional knowledge and 184 (44⋅2 %) got nutritional information. Results illustrated that health professionals were the primary source of nutritional information 130(70⋅7 %) (Table 4).

**Table 4.** Nutrition knowledge and information-related characteristics of adult cancer patients (≥18 years) at BLSH, Addis Ababa, Ethiopia, 2021 (*n* = 416)

Variables	Categories	Frequency	Percent
Nutritional knowledge	Good	269	64⋅7
	Poor	147	35⋅3
Nutritional information	Yes	184	44⋅2
	No	232	55⋅8
Source of information	Television	37	20⋅1
	Radio	8	4⋅3
	Health professionals	130	70⋅7
	Others^a^	9	4⋅9

a Internet, books, through experience sharing among patients.

### Food variety and meal frequency of the study participants

**Fig. 1.**
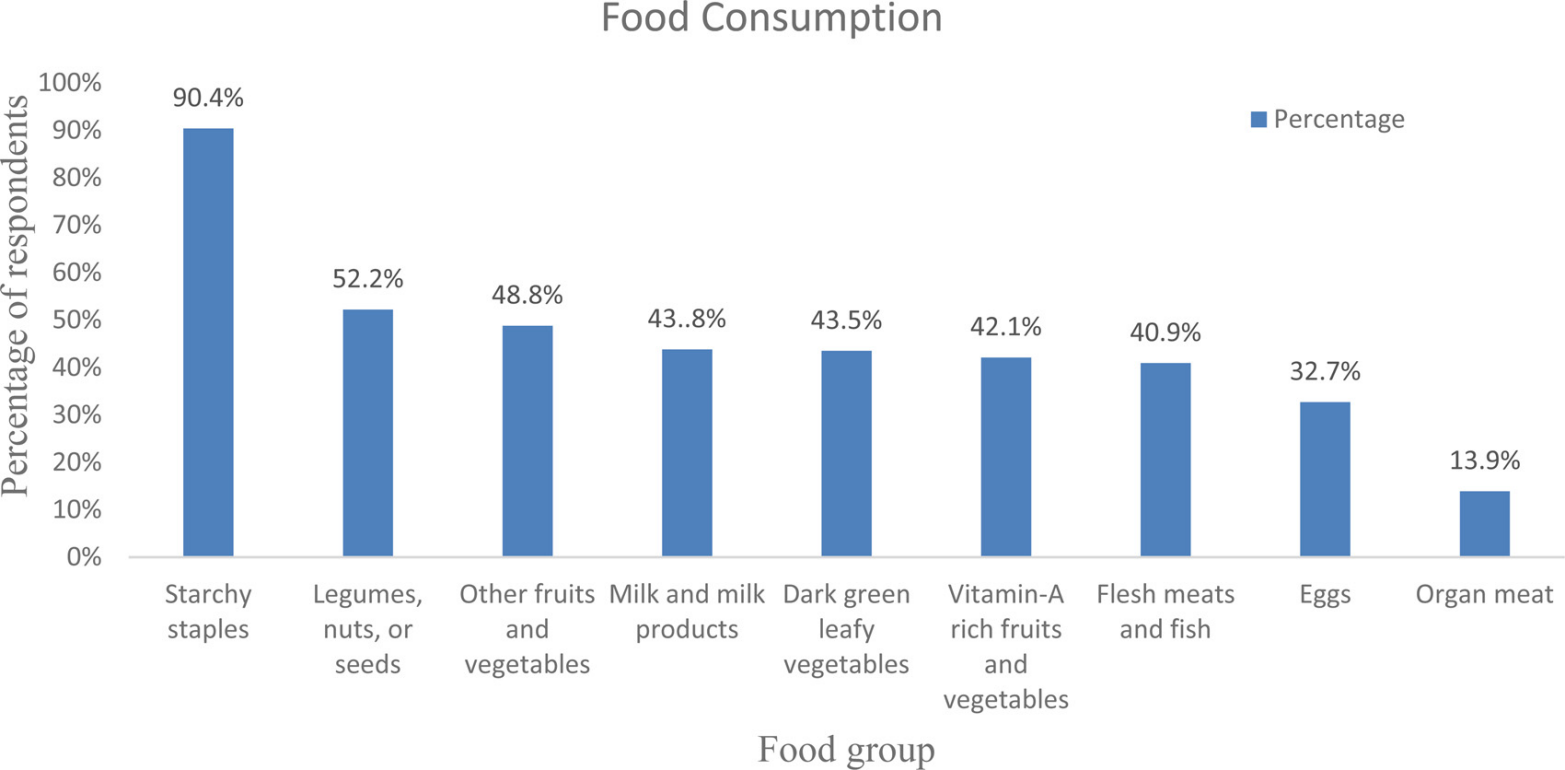
Consumption of diversified diet in 24 h before data collection among adult cancer patients (≥18 years) at BLSH, Addis Ababa, Ethiopia, 2021 (*n* = 416).

The daily average meal frequency was reported at 2⋅91(± 0⋅71). By considering the mean meal frequency as a cut-off point, three-hundred and nine (74⋅3 %) respondents had high meal frequency, i.e. ate three and more meals within the past 24 h before the data collection. As stated by the respondents, the most commonly consumed foods were starchy staples (90⋅4 %), oils and fats (79⋅3 %), legumes, nuts and seeds (52⋅2 %), and other fruits and vegetables (48⋅8 %). The food groups least consumed by the respondents were organ meat (13⋅9 %), egg (32⋅7 %) and flesh meat and fish (40⋅9 %) (Fig. 1).

### Dietary diversity and dietary pattern of study participants

**Fig. 2.**
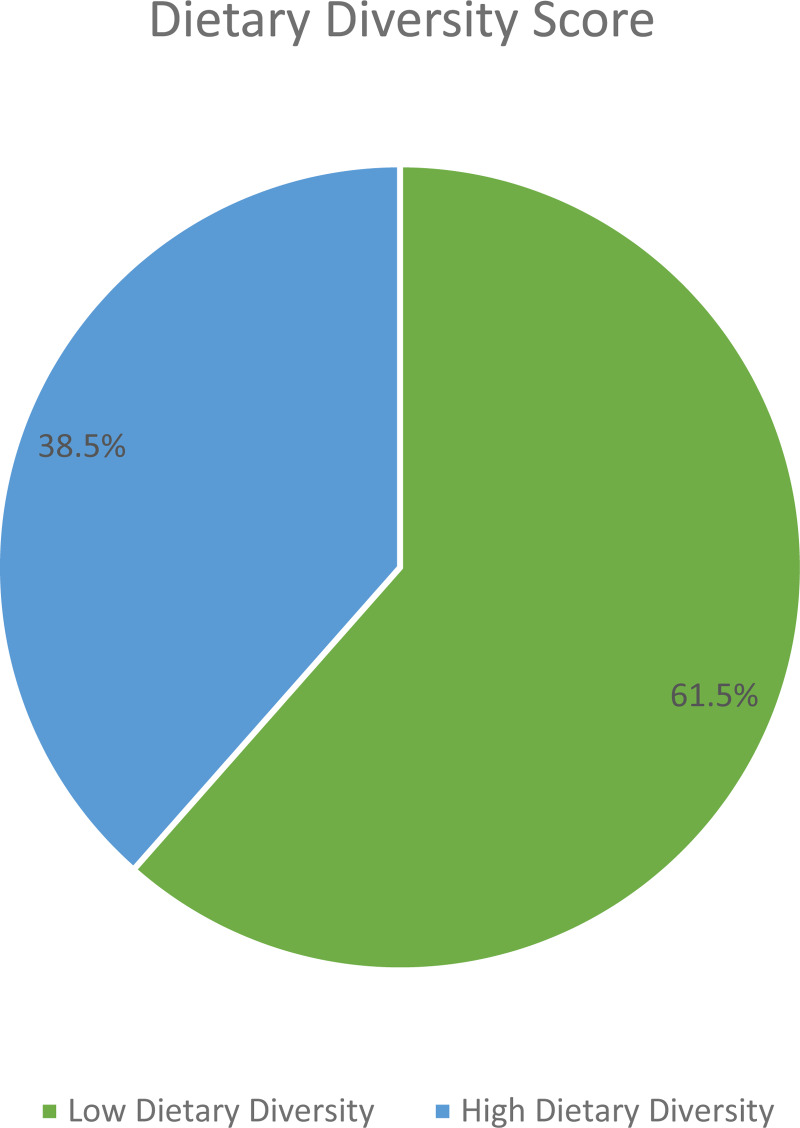
Dietary diversity status of adult cancer patients (≥18 years) at BLSH, Addis Ababa, Ethiopia, 2021 (*n* = 416).

The mean (±sd) IDDS was 4.08(±1⋅88). By considering the mean IDDS, about 61⋅5 % (CI 57⋅0, 66⋅0 %) of participants had low dietary diversity (Fig. 2).

### Factors associated with the level of dietary diversity

In bivariate logistic regression analysis, sex, age, residence, level of education, employment, family size, monthly income, alcohol consumption, regular physical exercise, year of diagnosis, treatment side effects, nutritional information, nutritional knowledge and meal frequency were associated with dietary diversity (*P* < 0⋅2). But after controlling for possible confounders, the result of the multi-variable analysis revealed that family size of ≥5, no permanent income, alcohol consumption of once a week, not doing regular physical activity, not having nutritional information, poor nutritional knowledge and meal frequency of <3 meals per day were factors significantly associated with poor dietary diversity of adult cancer patients (*P* < 0⋅05) (Table 5).

**Table 5.** Factors associated with dietary diversity of adult cancer patients (≥18 years) attending treatment at BLSH, Addis Ababa, Ethiopia, 2021 (*n* = 416).

Variables	Categories	Dietary Diversity		COR (95 % CI)	*P*-value	AOR (95 % CI)	*P*-value
		Low	High				
Sex	Male	109	47	1⋅78(1⋅17, 2⋅71)	0⋅007*	1⋅50(0⋅83, 2⋅70)	0⋅17
	Female	147	113	1		1	
Age	19–29	36	15	1⋅76(0⋅89, 3⋅48)	0⋅099*	1⋅38(0⋅52, 3⋅65)	0⋅51
	30–39	58	35	1⋅22(0⋅72, 2⋅05)	0⋅452	1⋅12(0⋅55, 2⋅28)	0⋅74
	40–49	67	40	1⋅23(0⋅75, 2⋅03)	0⋅408	1⋅45(0⋅76, 2⋅78)	0⋅25
	≥50	95	70	1		1	
Residence	Urban	153	125	1		1	
	Rural	103	35	2⋅40(1⋅53, 3⋅77)	<0⋅001*	1⋅51(0⋅79, 2⋅90)	0⋅21
Level of education	Unable to read and write	77	39	1⋅70(0⋅95, 3⋅04)	0⋅072*	1⋅34(0⋅52, 3⋅43)	0⋅54
	Read and write	32	14	1⋅97(0⋅92, 4⋅23)	0⋅081*	1⋅54(0⋅51, 4⋅62)	0⋅44
	Elementary	51	29	1⋅51(0⋅80, 2⋅85)	0⋅193*	1⋅61(0⋅68, 3⋅81)	0⋅27
	Secondary	52	40	1⋅12(0⋅61, 2⋅04)	0⋅705	0⋅78(0⋅36, 1⋅67)	0⋅52
	Collage and above	44	38	1		1	
Employment	Unemployed	164	90	1⋅38(0⋅92, 2⋅07)	0⋅112*	1⋅40(0⋅69, 2⋅82)	0⋅34
	Employed	92	70	1		1	
Family size	<5	130	61	1		1	
	≥5	126	99	0⋅59(0⋅39, 0⋅89)	0⋅012*	1⋅48(1⋅28, 1⋅83)	0⋅009**
Income	No permanent income	69	63	0⋅68(0⋅42, 1⋅10)	0⋅120*	1⋅31(1⋅15, 1⋅67)	0⋅003**
	≤3000 ETB	93	38	1⋅53(0⋅93, 2⋅52)	0⋅091*	0⋅92(0⋅47, 1⋅81)	0⋅82
	>3000 ETB	94	59	1		1	
Alcohol consumption	Not at all	197	149	1		1	
	Once a week	30	5	4⋅53(1⋅72, 11⋅9)	0⋅002*	3⋅97(1⋅20, 13⋅1)	0⋅02**
	Twice a week	19	4	3⋅59(1⋅19, 10⋅70	0⋅023*	2⋅65(0⋅68, 10⋅2)	0⋅15
	Three times and more	10	2	3⋅78(0⋅81, 17⋅5)	0⋅089*	3⋅70(0⋅54, 25⋅2)	0⋅18
Regular physical exercise	Yes	109	90	1		1	
	No	147	70	1⋅73(1⋅16, 2⋅58)	0⋅007*	1⋅83(1⋅07, 3⋅12)	0⋅02**
Year of Diagnosis	<1 year	89	66	1		1	
	1–3 years	96	52	1⋅36(0⋅86, 2⋅17)	0⋅185*	1⋅24(0⋅68, 2⋅25)	0⋅47
	3–5 years	46	26	1⋅31(0⋅73, 2⋅33)	0⋅356	1⋅74(0⋅83, 3⋅63)	0⋅13
	≥5 years	25	16	1⋅15(0⋅57, 2⋅34)	0⋅682	1⋅03(0⋅42, 2⋅48)	0⋅85
Loss of appetite	Yes	165	91	1⋅37(0⋅91, 2⋅06)	0⋅123*	1⋅06(0⋅59, 1⋅91)	0⋅83
	No	91	69	1		1	
Nausea	Yes	140	75	1⋅36(0⋅92, 2⋅03)	0⋅121*	0⋅74(0⋅35, 3⋅00)	0⋅42
	No	116	85	1		1	
Vomiting	Yes	91	46	1⋅36(0⋅89, 2⋅09)	0⋅152*	1⋅44(0⋅69, 3⋅07)	0⋅32
	No	165	114	1		1	
Nutritional information	Yes	93	91	1		1	
	No	163	69	2⋅31(1⋅54, 3⋅46)	<0⋅001*	2⋅23(1⋅30, 3⋅82)	0⋅003**
Nutritional knowledge	Good	141	128	1		1	
	Poor	115	32	3⋅26(2⋅06, 5⋅16)	<0⋅001*	1⋅84(1⋅05, 3⋅25)	0⋅03**
Meal Frequency	<3 meals	96	11	8⋅12(4⋅19, 15⋅7)	<0⋅001*	10⋅7(5⋅04, 22⋅7)	<0⋅001**
	3 meals	160	149	1		1	

*P-value < 0⋅2, **P-value < 0⋅05.

## Discussion

In a nutshell, the present study showed that around two-thirds of cancer patients had low dietary diversity. Family size, monthly income, alcohol consumption, regular physical activity, nutritional information, nutritional knowledge and meal frequency were significantly associated with low dietary diversity.

The overall magnitude of low dietary diversity in the present study was 61⋅5 % (CI 57⋅0, 66⋅0 %). Given the importance of IDDS in determining micronutrient adequacy of diet, this finding suggested that nearly two-thirds of adult cancer patients were at risk of having inadequate micronutrient intake^(1,23)^. The result is consistent with a study conducted in Jordan in which 64⋅3 % of cancer patients reported inadequate DDS^(25)^. However, the estimate in this study is higher compared to the study conducted in Kenya (37⋅7 %)^(24)^. This discrepancy could be attributable to the way the outcome variable was defined as well as the sampling method used.

According to the present study, starchy staples were the most predominantly (90⋅4 %) consumed food groups by the study participants. However, organ meats (13⋅8 %) were the least consumed food groups by the study participants in the study area. This might be due to economic problems, as organ meats are more expensive than starchy staples. Moreover, due to cultural pressures, people choose to purchase flesh meat rather than organ meat although organs are important micronutrient storage sites^(26)^. This finding is similar to various previous studies conducted on different study populations in the country and a similar studies in Kenya as well^(24,27,28)^.

The present study revealed an association between family size and low dietary diversity scores. Study participants who have a family size of five and more were 1⋅48 times more likely to have low dietary diversity than those who have a family size of below five. A plausible explanation for this finding is that when the number of family members increased, they will be unable to meet their family's financial needs. As a result, they devote more attention to meeting daily necessities than improving the quality of their nutrition^(29)^.

In line with the above explanation, the finding of the present study also revealed a positive association between dietary diversity and monthly income. Those who had no permanent monthly income were 1⋅31 times more likely to have low dietary diversity as compared with those who earn > 3000 ETB average monthly income. This result was also parallel with the previous studies in Libya^(30)^ and Columbus^(12)^. This might be, when an individual's income declines, so does their ability to purchase sufficient and diverse food.

Concerning the association between alcohol consumption and low DD of cancer patients, study participants who had the habit of drinking alcohol at least once a week were 3⋅97 times less likely to have a diversified diet than those who never drank. This may due to the fact that a wide range of hangover symptoms following alcohol intake like nausea, decreased appetite and inability to concentrate may cause reduced dietary intake and also alcohol may interfere with metabolism, and absorption of nutrients.

According to the present study, regular physical activity was significantly associated with dietary diversity. Cancer patients who had the habit of doing regular physical activity were 1⋅83 times more likely to have high dietary diversity than those who had no habit of doing regular physical activity. This could be attributed to the fact that participating in physical activities has been related to lower levels of tension, anxiety, and depression and boosting one's sense of wellness, and this might motivate individuals to follow healthy living in which dietary diversity practice belongs^(31)^.

Nutritional information was another predictor of DD among the study participants. Those adult cancer patients who got nutritional information were two times more likely to have a diversified diet compared with those who did not get nutritional information. This finding could be explained by the fact that as a person's nutritional knowledge grows, their willingness to try foods from various food groups also increases. As a result, a wide range of nutrients is obtained, resulting in nutrient adequacy^(32,33)^.

The present study also revealed that the nutritional knowledge of patients was significantly associated with dietary diversity. It was noted that cancer patients with poor nutritional knowledge were 1⋅8 times more likely to have low dietary diversity than those having good nutritional knowledge. This finding was consistent with the study finding in Kenya^(24)^ and Jordan^(25)^ and studies conducted in Bahirdar among HIV patients and pregnant women^(34,35)^. This could be because good nutritional knowledge is one of the requirements for good dietary diversity practice.

The findings of the present study also revealed that the meal frequency of patients was significantly associated with dietary diversity. Those patients having a meal frequency of fewer than three times a day were ten times more likely to have a non-diversified diet than those having a meal frequency of three and more times. This finding is in line with the study finding in Brazil, in which eating frequency was positively associated with diet quality^(36)^. This could be explained by the fact that increasing the frequency of consuming food items is one of the strategies to increase the DD of individuals.

Although the present study addressed very significant dietary diversity variables, information regarding food serving size and nutritionally related biochemical parameters were left out, making it impossible to directly infer from the results whether an individual's micronutrient intake is adequate. Even though using a 24-h dietary recall method reduces recall bias, it does not show the usual dietary habit of patients. The present study is also not independent of selection bias.

## Conclusion

Overall, the present study showed that the majority of cancer patients had low DD, suggesting that they are highly vulnerable to micronutrient deficiencies^(14)^. High family size, having no permanent income, alcohol consumption, not doing regular physical exercise, lack of nutritional information, poor nutritional knowledge and minimal meal frequency were factors significantly associated with low DD among adult cancer patients. Therefore, efforts should be strengthened to improve patients’ income level, access to nutrition information and nutritional knowledge.
